# Genetics impact risk of Alzheimer’s disease through mechanisms modulating structural brain morphology in late life

**DOI:** 10.1136/jnnp-2023-332969

**Published:** 2024-04-25

**Authors:** Roxanna Korologou-Linden, Bing Xu, Elizabeth Coulthard, Esther Walton, Alfie Wearn, Gibran Hemani, Tonya White, Charlotte Cecil, Tamsin Sharp, Henning Tiemeier, Tobias Banaschewski, Arun Bokde, Sylvane Desrivières, Herta Flor, Antoine Grigis, Hugh Garavan, Penny Gowland, Andreas Heinz, Rüdiger Brühl, Jean-Luc Martinot, Marie-Laure Paillère Martinot, Eric Artiges, Frauke Nees, Dimitri Papadopoulos Orfanos, Tomáš Paus, Luise Poustka, Sabina Millenet, Juliane H Fröhner, Michael Smolka, Henrik Walter, Jeanne Winterer, Robert Whelan, Gunter Schumann, Laura D Howe, Yoav Ben-Shlomo, Neil M Davies, Emma Louise Anderson

**Affiliations:** 1 Medical Research Council Integrative Epidemiology Unit, University of Bristol, Bristol, UK; 2 Population Health Sciences, University of Bristol, Bristol, UK; 3 The Generation R Study Group, Erasmus MC University Medical Center, Rotterdam, UK; 4 Department of Child and Adolescent Psychiatry and Psychology, Erasmus MC University Medical Center, Rotterdam, The Netherlands; 5 Bristol Medical School, University of Bristol, Bristol, UK; 6 North Bristol NHS Trust, Bristol, UK; 7 Department of Psychology, University of Bath, Bath, UK; 8 Department of Radiology and Nuclear Medicine, Erasmus University School of Medicine, Rotterdam, UK; 9 Department of Epidemiology, Erasmus MC University Medical Center Rotterdam, Rotterdam, The Netherlands; 10 Biostatistics and Health Informatics Department, King’s College London, Boston, UK; 11 Department of Social and Behavioral Sciences, Harvard T H Chan School of Public Health, Boston, Massachusetts, USA; 12 Department of Child and Adolescent Psychiatry and Psychotherapy, Heidelberg University, Heidelberg, Germany; 13 Psychiatry, Trinity College Dublin, Dublin, Ireland; 14 Kings College London, Centre for Population Neuroscience and Precision Medicine (PONS), London, UK; 15 Institute of Cognitive and Clinical Neuroscience, Central Institute of Mental Health, University of Mannheim, Mannheim, Germany; 16 Psychology, School of Social Sciences, University of Mannheim, Mannheim, Germany; 17 Neurospin, LNAO, I2BM, CEA, Saclay, France; 18 University of Vermont, Burlington, Vermont, USA; 19 University of Nottingham, Nottingham, UK; 20 Department of Psychiatry and Psychotherapy CCM, Berlin Institute of Health, Berlin, Germany; 21 Physikalisch-Technische Bundesanstalt (PTB), Braunschweig, Germany; 22 Institut National de la Santé et de la Recherche Médicale, INSERM U1299, Paris, France; 23 Centre Borelli, Cachan, France; 24 Institute of Medical Psychology and Medical Sociology, Kiel University, Kiel, Germany; 25 NeuroSpin, Université Paris-Saclay, Gif-sur-Yvette, France; 26 Departments of Psychology and Psychiatry, University of Toronto, Toronto, Ontario, Canada; 27 Department of Psychiatry, University of Montreal, Montreal, Quebec, Canada; 28 Department of Child and Adolescent Psychiatry and Psychotherapy, University Medical Centre Göttingen, Göttingen, Germany; 29 Department of Psychiatry, Technische Universität Dresden, Dresden, Germany; 30 Department of Psychiatry and Psychotherapy CCM, Charité Universitätsmedizin Berlin, Berlin, Germany; 31 Berlin Institute of Health at Charite, Berlin, Germany; 32 Department of Education and Psychology, Freie Universität Berlin, Berlin, Germany; 33 Trinity Centre for Bioengineering, Trinity College Dublin, Dublin, Ireland; 34 Fudan University, Shanghai, People's Republic of China; 35 PONS Centre, Dept. of Psychiatry and Clinical Neuroscience, CCM, Berlin, Germany; 36 University College London Division of Psychiatry, London, UK

**Keywords:** Alzheimer's disease, genetics, epidemiology, neuroanatomy, brain mapping

## Abstract

**Background:**

Alzheimer’s disease (AD)-related neuropathological changes can occur decades before clinical symptoms. We aimed to investigate whether neurodevelopment and/or neurodegeneration affects the risk of AD, through reducing structural brain reserve and/or increasing brain atrophy, respectively.

**Methods:**

We used bidirectional two-sample Mendelian randomisation to estimate the effects between genetic liability to AD and global and regional cortical thickness, estimated total intracranial volume, volume of subcortical structures and total white matter in 37 680 participants aged 8–81 years across 5 independent cohorts (Adolescent Brain Cognitive Development, Generation R, IMAGEN, Avon Longitudinal Study of Parents and Children and UK Biobank). We also examined the effects of global and regional cortical thickness and subcortical volumes from the Enhancing NeuroImaging Genetics through Meta‐Analysis (ENIGMA) Consortium on AD risk in up to 37 741 participants.

**Results:**

Our findings show that AD risk alleles have an age-dependent effect on a range of cortical and subcortical brain measures that starts in mid-life, in non-clinical populations. Evidence for such effects across childhood and young adulthood is weak. Some of the identified structures are not typically implicated in AD, such as those in the striatum (eg, thalamus), with consistent effects from childhood to late adulthood. There was little evidence to suggest brain morphology alters AD risk.

**Conclusions:**

Genetic liability to AD is likely to affect risk of AD primarily through mechanisms affecting indicators of brain morphology in later life, rather than structural brain reserve. Future studies with repeated measures are required for a better understanding and certainty of the mechanisms at play.

**WHAT IS ALREADY KNOWN ON THIS TOPIC**

- Little is known about the dynamic interplay between brain morphology and Alzheimer’s disease throughout the life course.
- Most prior research has predominantly focused on overall brain structure metrics, such as estimated total intracranial volume, mean thickness and total surface area.

**WHAT THIS STUDY ADDS**

- This is the first bidirectional Mendelian randomisation study to assess the effects between Alzheimer’s disease, and both global and regional measurements of cortical thickness, estimated total intracranial volume, total white matter and subcortical structure volumes, using different cohorts spanning the life course.

**HOW THIS STUDY MIGHT AFFECT RESEARCH, PRACTICE OR POLICY**

- Brain morphology is likely to play a role in changing the risk of Alzheimer’s disease through neurodegenerative pathways such as a loss of brain matter, rather than neurodevelopmental pathways such as building brain reserve.
- Future research should focus efforts on using different measures of structural and functional brain morphology, starting in mid-adulthood.

## Introduction

 The earliest Alzheimer’s disease (AD)-related histopathological changes are typically observed within the medial temporal lobes and disperse throughout the frontal, parietal and temporal neocortices and subcortical regions by the time a clinical diagnosis of AD is made.^1^ Amyloid-β accumulation in the brain may be apparent 20 years before the appearance of clinical symptoms.^2^ Hence, the integration of biological data prior to the onset of clinical symptoms is crucial in understanding the aetiology, timing and progression of the disease, and for the development of more efficient strategies for early detection and screening of individuals for AD risk.

It has been argued that variability in AD risk may be mediated through both morphology (‘brain reserve’) and/or functional capacity to compensate for pathology (‘cognitive reserve’),^3^ which may operate independently or synergistically. Consequently, it has been hypothesised that genetic risk for AD may be mediated through determining the underlying brain reserve of an individual.^4^,^6^ Furthermore, the relationship between brain structures and AD may be bidirectional, as genes associated with brain morphology (such as thickness and surface area) have been shown to be involved in neurogenesis.^7^

Genetic instruments allow for the identification of factors that modify disease risk, establish effects of prodromal disease and can help us discover biomarkers that predict disease. Genome-wide association studies (GWAS)^8^,^11^ for AD have identified approximately 30 single nucleotide polymorphisms (SNPs), each with a modest effect on the risk of AD, apart from the ε4 genotype in the *APOE* gene, whereby carriers may have up to 12-fold increased risk.^11^ Previous studies examining the association of genetic liability to AD with brain morphology have typically used polygenic risk scores (PRS) at liberal thresholds, which can increase bias due to horizontal pleiotropy.^6 12 13^ They also have small sample sizes, as genetic and neuroimaging data are rarely available in combination. Furthermore, SNPs associated with brain structure have been discovered using larger sample sizes^7 14^ than previous neuroimaging studies,^15 16^ allowing for the bidirectional investigation of the causal effects of structural brain measures on risk of AD, using Mendelian randomisation (MR). MR is a form of instrumental variable analysis which uses SNPs as instruments for exposures to estimate lifetime effects of phenotypes on disease risk (and vice versa).^17^

We investigated how genetic liability to AD affects brain morphology across the life course (from ages 8 to 81 years) using two-sample MR. This approach examines whether AD genetic susceptibility affects brain development or degeneration. Using two-sample MR, we also investigated whether brain morphology has a causal effect on the risk of AD, to establish whether greater thickness/volume provides a protective effect against advancing neuropathology and thus, reduces risk of an AD diagnosis (‘brain reserve’ hypothesis).

## Materials and methods

### Data

#### Alzheimer’s disease GWAS

We extracted SNPs from the largest GWAS of clinically diagnosed AD,^9^ which identified 27 SNPs to be associated with AD risk in participants of European ancestry. For each SNP, we used the effect estimates from the stage with the largest sample size (n=82 771 to 94 437 participants).

#### Brain structure GWAS

**Figure 1:**
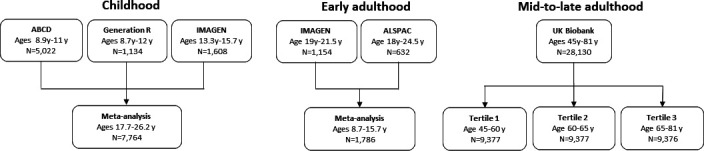
Study cohorts in the age-stratified analysis of genetic liability to Alzheimer’s disease on brain morphology. ABCD, Adolescent Brain Cognitive Development; ALSPAC Avon Longitudinal Study of Parents and Children; y, years.

We used GWAS of brain structures (average thickness of 34 gyral-based cortical regions of interest, mean thickness, estimated total intracranial volume (eTIV), 9 subcortical volumes and the total volume of white matter) conducted within different cohorts across the life course. Regional thickness has been used to differentiate between mild cognitive impairment and individuals with AD with excellent accuracy, specificity and reproducibility across independent cohorts.^18^ We conducted all GWAS described, except for the GWAS in the ENIGMA consortium which has been previously published.^7 14 19 20^ GWAS for regional cortical thickness and subcortical volumes were adjusted for global cortical thickness and eTIV, respectively. For the peri-pubertal period, we used Generation R,^21 22^ the Adolescent Brain Cognitive Development study (ABCD)^23 24^ and IMAGEN.^25^ For early adulthood, we meta-analysed the Avon Longitudinal Study of Parents and Children (ALSPAC)^26^,^28^ and the second wave of data collection for the IMAGEN study.^25^ For adulthood, we used the UK Biobank (UKB)^29^ and stratified the sample into three equal-sized age tertiles, to examine age-specific effects (figures1  2 and table 1). Finally, we used summary data from ENIGMA^7 14^ (n=37 741 participants, age range 3.4–91.4 years), which includes the first release of UKB imaging data. All GWAS in the analyses were conducted in participants of European ancestry. Details of the cohorts, including the genotyping and neuroimaging procedures, are provided in online supplemental tables 1 and 2, respectively.

**Figure 2:**
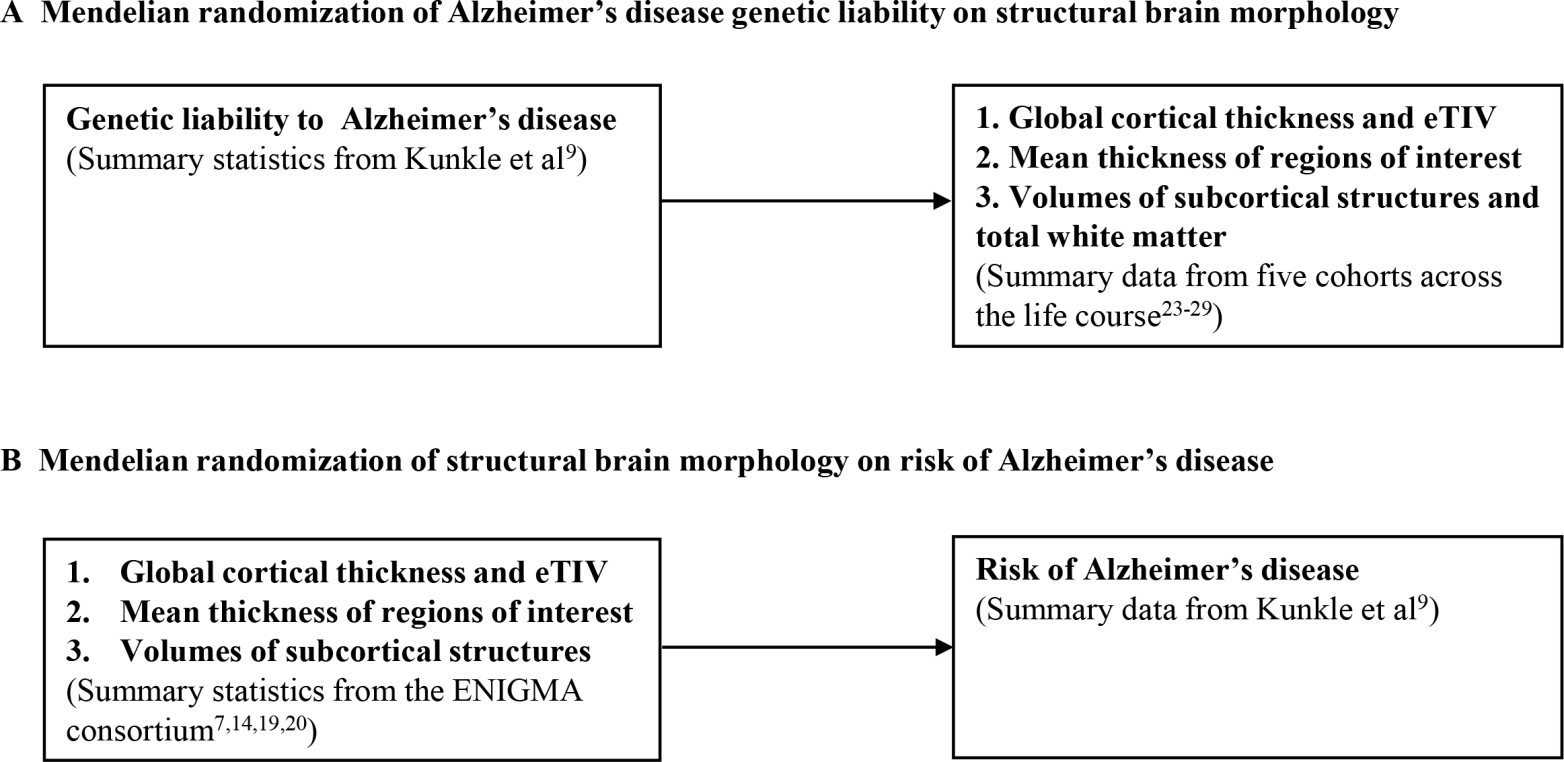
Study design for examining the bidirectional effects between Alzheimer’s disease and brain morphology. (A) Mendelian randomisation of Alzheimer’s disease genetic liability on structural brain morphology. (B) Mendelian randomisation of structural brain morphology on risk of Alzheimer’s disease. eTIV, estimated total intracranial volume.

**Table 1:** Descriptive statistics of the cohorts used in the analysis

Cohort	N	Number of Alzheimer’s disease SNPs	F-statistic for Alzheimer’s disease SNPs	Mean age (SD)	Age range	% female
Childhood						
ABCD	5022	25	223.28	9.91 (0.6)	8.9–11	52.6
Generation R	1134	23	239.34	10.2 (0.6)	8.9–12	49.2
IMAGEN	1151–1154	23	224.67	14.4 (0.4)	13.3–15.7	50.6
Early adulthood						
ALSPAC	358–632	25	231.7	20.5 (1.6)	18–24.5	22.4
IMAGEN	1577–1608	23	224.7	26.2 (0.7)	17.7–26.2	51.3
Adulthood						
UK Biobank tertile 1	9377	24	231.5	55 (3.4)	45–60	57.0
UK Biobank tertile 2	9377	24	231.5	64.3 (2.2)	60–68	53.7
UK Biobank tertile 3	9376	24	231.5	72.0 (2.9)	68–81	46.0

ABCD, Adolescent Brain Cognitive Development; ALSPAC, Avon Longitudinal Study of Parents and Children; SNP, single nucleotide polymorphism.

### Statistical analyses

#### Estimating the causal effect of genetic liability to Alzheimer’s disease on brain structures

##### Two-sample Mendelian randomisation

Two-sample MR is an extension of MR,^30^ where the SNP effects on the exposure and on the outcome are extracted from separate GWAS studies. To examine the effects of genetic liability to AD on structural brain measures, we extracted SNPs strongly associated with AD (p≤5×10^–8^).^9^ Where SNPs were not available, we used proxy SNPs (r^2^>0.80). SNPs were clumped using r^2^>0.001 and a physical distance of 10 000 kb. We also included rs7412 and rs423958 to tag the *APOE* ε4 allele. We used 23–25 SNPs as instruments for AD, the number varying by availability within each cohort (table 1). We harmonised the AD and brain structure GWAS in IMAGEN, Generation R, ABCD, ALSPAC and the UKB (online supplemental methods). We then employed univariable MR to estimate the effect of the AD SNPs on 9 subcortical volumes and the 34 cortical regions defined by the Desikan-Killiany atlas^31^ (as well as total volume of white matter where available) within each cohort. We used a random-effects inverse-variance weighted (IVW) regression analysis, which assumes no directional horizontal pleiotropy^17^ and used the F-statistic as a measure of instrument strength.^32^ All effect estimates reflect SD changes in the outcome per doubling of genetic liability to AD.^33^ Using the metagen function,^34^ we applied random-effects models to meta-analyse the effects of the AD SNPs on structural brain measures for the three peri-pubertal cohorts: IMAGEN, ABCD and Generation R (figures1  2). To examine how the age-level covariate was associated with the causal effect estimates across the three age-stratified tertiles of UKB, we extracted a p for trend across these groups, using the meta regress command in STATA V.16^35^ and using the mean age of each tertile as the exposure. Sample sizes differed by brain structure due to quality control and missing data.

### Estimating the causal effect of brain structures on risk of Alzheimer’s disease

#### Two-sample Mendelian randomisation

Using the ENIGMA GWAS,^7 14 19 20^ we extracted SNPs associated with eight subcortical volumes and the thickness of the regions of interest as defined by the Desikan-Killiany atlas.^31^ The same parameters and harmonisation methods were used as in the previous analysis. Again, we employed univariable MR to examine the causal effects of each brain structure on risk of AD using a random-effects IVW regression. All effect estimates represent an OR for AD per SD increase in thickness or volume. There is overlap between ENIGMA and some of the individual-level cohorts. However, it has been shown that sample overlap results in little bias in the presence of strong instruments (ie, F>10).^36^

#### Sensitivity analyses

We conducted sensitivity analyses to examine for potential violation of key MR assumptions. For MR to generate unbiased causal effect estimates, each genetic variant that is used as an instrumental variable must satisfy three assumptions: (1) that it is associated with the exposure (relevance assumption), (2) that it is not associated with the outcome through a confounding pathway (exchangeability assumption) and (3) is only associated with the outcome through the exposure (exclusion restriction assumption). IVW regression assumes no horizontal pleiotropy and provides unbiased causal effect estimates only when there is balanced or no horizontal pleiotropy. We compared estimates from IVW with those from Egger regression,^37 38^ weighted median^39^ and weighted mode,^40^ which relax this assumption. Heterogeneity in the causal estimates was assessed using Cochran’s Q-statistic.^37^ Furthermore, to exclude the possibility that the SNPs used to proxy for AD are instruments for brain structures and vice versa, we performed a directionality (Steiger) test.^41^ Where the hypothesised direction was false, we performed sensitivity analyses removing SNPs explaining more variance in the outcome than the exposure (details in online supplemental methods). Lastly, we excluded the two SNPs in the *APOE* locus from the AD instrument, to investigate whether the effects observed are primarily driven by the variants in the *APOE* gene. This study involves evaluating global patterns of effect estimates; hence, we focus on effect size and precision.^42 43^ Adjusted p values, controlling for the false discovery rate are in provided online supplemental tables 4, 8, 9 and 11.

## Results

We used bidirectional two-sample MR^30^ to first examine the effect of genetic liability to AD (p≤5×10^−8^) on global and regional cortical thickness, eTIV, volumes of subcortical structures. We also included total white matter as an outcome where available. To boost the statistical power of the smaller childhood cohorts, we meta-analysed the causal effect estimates across ABCD,^23 24^ Generation R^44 45^ and IMAGEN^25^ (aged 8–16 years). For early adulthood, we used participants selected for neuroimaging in ALSPAC substudies^46^ (aged 18–24.5 years). For mid-to-late adulthood, we stratified the UKB population into three age tertiles: 45–60 years, 60–68 years and 68–81 years. In total, we used 23–25 independent AD SNPs from the largest GWAS of clinically diagnosed AD,^9^ depending how many were available in each cohort used (table 1). Second, we examined the causal effects of brain morphology on AD risk, using genetic instruments for each brain structure from the ENIGMA consortium GWAS. A summary of our study design is presented in figures1  2.

Of the 34 cortical regions and 10 subcortical structures examined, there was evidence to suggest that genetic liability to AD has an age-dependent effect on the thickness and volume of these measures, respectively, across mid-to-late adulthood, but the evidence for such effects in childhood through young adulthood is weak. When we examined the causal effects of the thickness of 27 cortical regions (ie, those regions with genetic variants at 5×10^–8^), we found little evidence of an effect of greater thickness on risk of AD. We only found evidence that hippocampal volume and thickness of lateral orbitofrontal and rostral anterior cingulate cortices affected AD risk. An overview of the findings is shown in table 2.

**Table 2:** Summary of main findings

Exposure	Outcome	Timepoint	Direction
Genetic liability to Alzheimer’s disease	Caudal anterior cingulate	Childhood	↓
	Thalamus		↓
	Thalamus	Early adulthood	↓
	Cuneus	Adulthood (45–60 years)	↑
	Inferior temporal		↑
	Accumbens	Adulthood (60–68 years)	↓
	Caudal middle frontal		↓
	Caudate		↓
	Putamen		↓
	Thalamus		↓
	Accumbens*	Adulthood (68–81 years)	↓
	Amygdala*		↓
	Caudate		↓
	Entorhinal*		↓
	Fusiform		↓
	Hippocampus*		↓
	Inferior temporal		↓
	Lateral occipital		↑
	Lateral ventricles		↑
	Middle temporal*		↓
	Parahippocampal		↓
	Pericalcarine		↑
	Postcentral		↑
	Precentral		↑
	Superior parietal		↑
	Thalamus*		↓
	Transverse temporal		↑
Hippocampus	Alzheimer’s disease	Across the life course (summary data)	↑

Only analyses where 95% confidence intervalCIs show some evidence of association are displayed.

* Indicates p<0.05 following correction for multiple testing.

### Causal effects of genetic liability to Alzheimer’s disease on brain structures

#### Childhood

**Figure 3:**
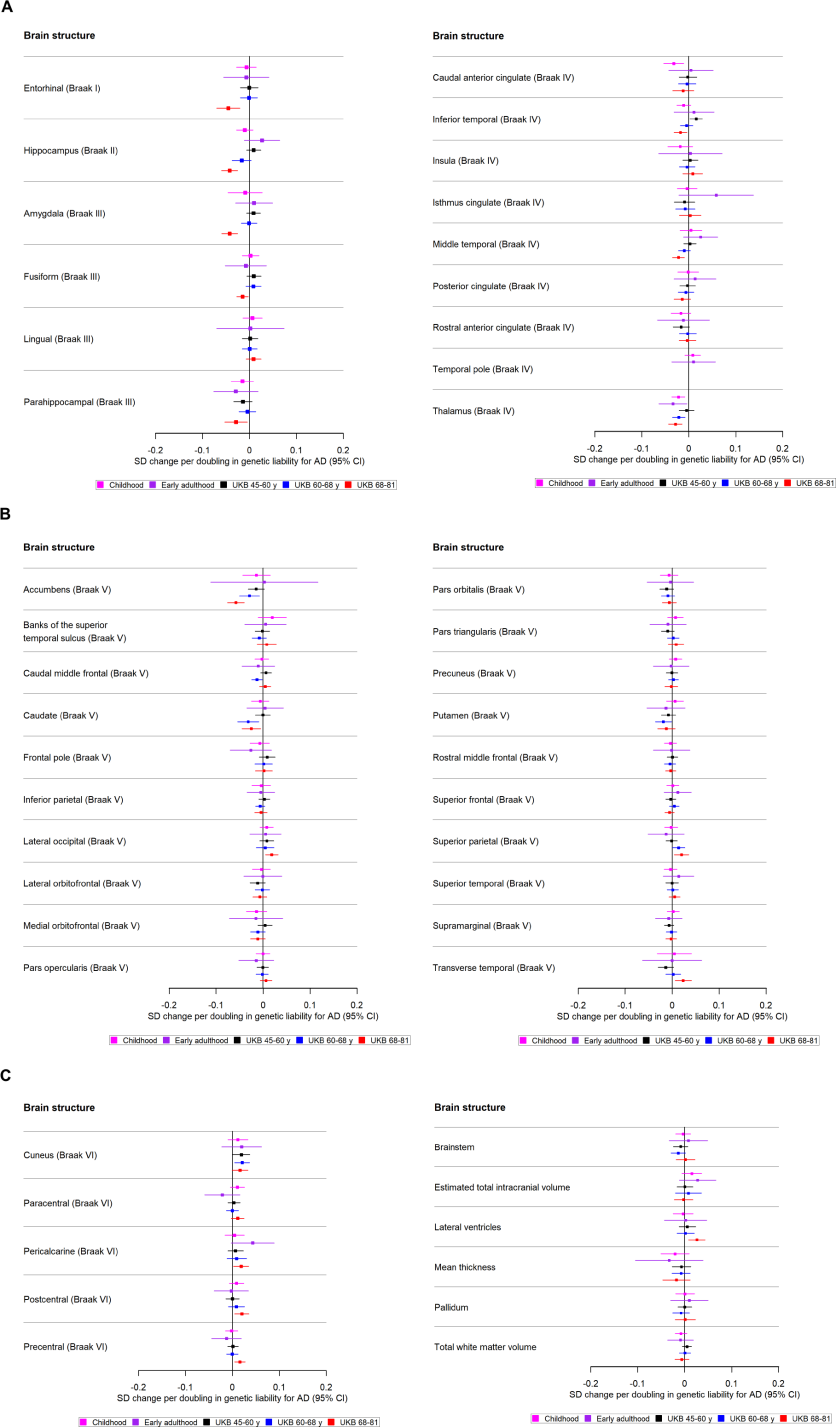
(**A**) The causal effects of genetic liability to AD on brain structures in Braak stages I–IV at different ages across the life course (see figure 3B for structures in Braak stage V and figure 3C for Braak stage VI). The childhood cohorts include meta-analysed effects of three peri-pubertal cohorts: ABCD, GEN R and IMAGEN. The early adulthood cohort includes ALSPAC and the later adulthood cohorts include UKB. Effect estimates for cortical regions and subcortical structures represent SD changes in thickness and volume. Cortical regions were adjusted for mean thickness and subcortical volumes were adjusted for estimated intracranial volume. Where an effect estimate is missing, that structural measure was not available in that cohort. (**B**) The causal effects of genetic liability to AD on brain structures in Braak stages V at different ages across the life course (see figure 3C for structures in Braak stage VI). The childhood cohorts include meta-analysed effects of three peri-pubertal cohorts: ABCD, GEN R and IMAGEN. The early adulthood cohort includes ALSPAC and the later adulthood cohorts include UKB. Effect estimates for cortical regions and subcortical structures represent SD changes in thickness and volume. Cortical regions were adjusted for mean thickness and subcortical volumes were adjusted for estimated intracranial volume. Where an effect estimate is missing, that structural measure was not available in that cohort. (**C**) The causal effects of genetic liability to AD on brain structures in Braak stage VI, and those not included in Braak staging, at different ages across the life course. The childhood cohorts include meta-analysed effects of three peri-pubertal cohorts: ABCD, GEN R and IMAGEN. The early adulthood cohort includes ALSPAC and the later adulthood cohorts include UKB. Effect estimates for cortical regions and subcortical structures represent SD changes in thickness and volume. Cortical regions were adjusted for mean thickness, subcortical structures and volume of cerebral white matter were adjusted for estimated intracranial volume. Where an effect estimate is missing, that structural measure was not available in that cohort. ABCD, Adolescent Brain Cognitive Development; AD, Alzheimer’s disease; ALSPAC, Avon Longitudinal Study of Parents and Children; GEN R, Generation R; UKB, UK Biobank.

Only weak evidence supported the association between genetic liability to AD and cortical thickness or subcortical volumes in school-aged children. A doubling in odds of genetic liability to AD was associated with a −0.02 SD (95% CI −0.04 to –0.01) smaller volume of the thalamus (Braak stage IV) (figure 3A) and −0.03 SD (95% CI −0.05 to –0.01) lower thickness of the caudal anterior cingulate (Braak stage IV) (figure 3A, online supplemental tables 1–4).

#### Early adulthood

There was little evidence to suggest that a higher genetic liability to AD is associated with cortical regions and subcortical structures. However, a doubling in odds of genetic liability to AD was weakly associated with a −0.03 lower thalamic volume (Braak stage IV) (figure 3A, online supplemental table 8) (SD−0.03; 95% CI −0.06 to –0.004).

#### Mid-life to late life

We identified evidence of an age-dependent effect of AD genetic liability on smaller volume of the hippocampus (Braak stage II), accumbens (Braak stage II), amygdala (Braak stage II) and thalamus (Braak stage IV) (p for trend across age tertiles for each respective structure: 1.32×10^–5^, 0.001, 0.02 and 0.03; online supplemental tables 9 and 10). Furthermore, we found evidence of age-dependent effect of AD genetic liability on lower thickness of the inferior temporal and middle temporal cortices (p for trend across age tertiles=0.001 and p=0.009, respectively; Braak stage IV, figure 3A). A doubling in odds of genetic liability to AD, for example, was associated with 0.02 SD (95% CI −0.04 to –0.01) lower thickness in the middle temporal cortex for participants of aged 68–81 years and a trend in the same direction was observed for participants aged 60–68 years. On the contrary, for the superior and transverse temporal cortices (Braak stage V, figure 3B), we identified AD genetic liability to be associated with greater thickness (p for trend across age tertiles=0.03 and p=0.003, respectively).

We also identified effects which did not show clear age-dependent associations. Within the youngest UKB participants aged 45–60 years, a higher genetic liability to AD was associated with a greater thickness in the cuneus. In participants aged 60–68 years, a higher genetic liability to AD was associated with a lower volume in the caudate (Braak stage V, figure 3B), and putamen (Braak stage V, figure 3B). In participants aged 68–81 years, a doubling in odds of genetic liability to AD was associated with 0.05 SD (95% CI 0.07 to 0.02) lower thickness in the entorhinal cortex (Braak stage I), fusiform and parahippocampal cortices (Braak stage III, figure 3A). Additionally, AD genetic liability was associated with a thicker pericalcarine, postcentral, precentral cortex and a larger volume in the lateral ventricles (Braak stage VI, figure 3C).

#### Causal effects of brain morphology on risk of Alzheimer’s disease

**Figure 4:**
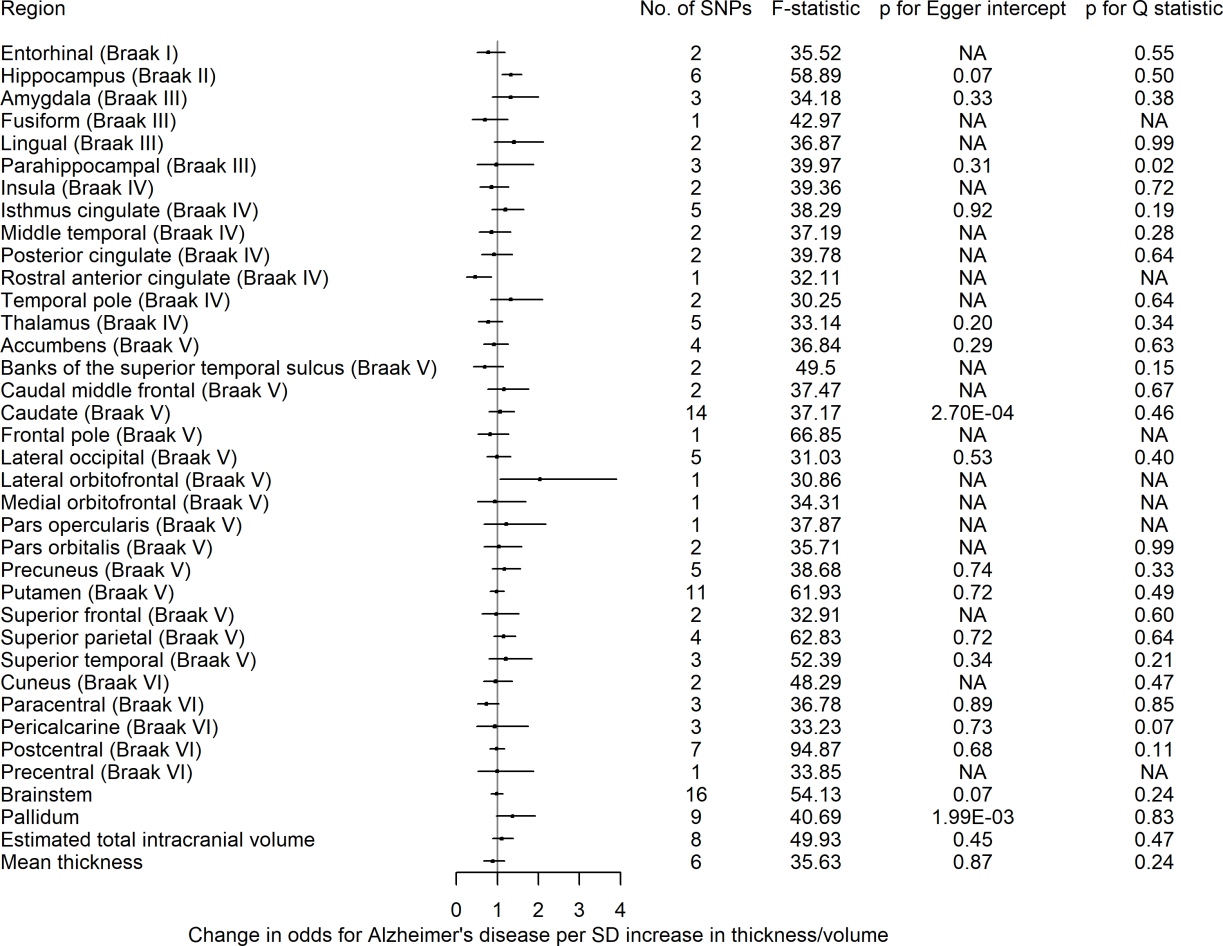
The causal effects of genetic predisposition to higher thickness and volume of cortical, subcortical and white matter measures, respectively on risk for AD. This figure shows the change in OR for AD per SD change in thickness and volume of cortical, subcortical structures, respectively. Effects for lateral ventricles is missing due to inability in obtaining access to summary statistics. The F-statistic is a measure of instrument strength. AD, Alzheimer’s disease; NA, not available.

We found little evidence of causal effects for the global measures of thickness and eTIV on AD risk (online supplemental table 11). However, of the eight subcortical structures examined, we observed that a 1 SD increase hippocampal volume, instrumented by six SNPs, increased AD risk on average by 33% (95% CI 1.11 to 1.59). A 1 SD increase in the thickness of the lateral orbitofrontal cortex increased AD risk (OR 2.74; 95% CI 1.08 to 6.93), while a 1 SD higher thickness in the rostral anterior cingulate cortex decreased AD risk (OR 0.40; 95% CI 0.19 to 0.83) (figure 4). However, for these two structures, we have one instrument and could not perform sensitivity analyses for assessing heterogeneity or pleiotropy.

#### Sensitivity analyses

Detailed results of analyses examining potential pleiotropy are provided in online supplemental tables 1–18. The evidence of a causal effect of genetic liability to AD on the caudal anterior cingulate in peri-pubertal childhood was consistent across pleiotropy-robust methods (SD −0.03; 95% CI −0.06 to –0.004 in MR-Egger and SD −0.03; 95% CI −0.05 to –0.01 per doubling in odds of genetic liability to AD for weighted mode). The association with thalamic volume in school-aged children and early adulthood were consistent across most of the pleiotropy-robust methods (online supplemental tables 4 and 8). In the analysis of AD genetic liability on brain structures in UKB, the magnitude of effect sizes for the MR-Egger, weighted median and mode were consistent with the IVW estimates for all brain structures (online supplemental table 9). In the MR analysis of brain structures on AD risk, the observed detrimental effect of a larger hippocampus on AD was consistent across pleiotropy-robust methods (online supplemental tables 11 and 18).

When we removed the *APOE* SNPs from our analyses in the peri-pubertal childhood cohort meta-analysis, the effect observed for AD liability on thalamic volume and the thickness of the caudal anterior cingulate cortex attenuated to the null (online supplemental table 19). The effect observed for AD liability on thalamic volume in early adulthood also attenuated to the null (online supplemental table 20). In UKB analyses, the associations with regional cortical thickness and subcortical structures largely remained (online supplemental table 21).

The directionality test indicated that, on average, the instruments for AD explained more variance in AD than they did in the brain structures in UKB (online supplemental table 22). The directionality test for SNPs associated with the hippocampus, lateral occipital and rostral anterior cingulate cortices on AD showed that they explain more variance in these structures than they do in AD risk (online supplemental table 23).

## Discussion

Our findings suggest that AD risk alleles have an age-dependent effect on a range of cortical and subcortical brain measures across mid-to-late adulthood, but we found little evidence for such effects in childhood and early adulthood, with the exception of an observed effect of AD genetic liability on thalamic volume and the thickness of the caudal anterior cingulate. These results therefore suggest that genetic liability to AD operates largely through causing changes in brain morphology in later life (eg, potential neurodegeneration), rather than initial brain reserve. In the age-stratified analysis of UKB participants, a higher AD genetic liability was associated with an age-dependent decrease in the thickness of the middle temporal, inferior temporal cortices as well as volume of structures such as the hippocampus, accumbens and thalamus. Some effects were only apparent in the oldest participants (aged 68–81 years), such as the decrease in the thickness of the fusiform, entorhinal and parahippocampal cortices and the volume of the amygdala. When SNPs in the *APOE* gene region were removed, effects across all structures largely remained but as expected, became less precise. In the reverse direction, there was little evidence that the thickness and volume of cortical and subcortical structures, respectively, affected the risk of AD, except for a greater hippocampal volume increasing risk.

In adults, genetic liability to AD was associated with regions known to show significant atrophy early in disease progression, such as the entorhinal,^47^,^49^ inferior, middle temporal and parahippocampal cortices,^50^ as well as the hippocampus.^51^ Change in hippocampal volume is an important imaging phenotype to define preclinical stages of AD, where atrophy predicts conversion from mild cognitive impairment to AD.^52^ We observed a trend of a higher genetic liability to AD being associated with a smaller hippocampus in the younger participants, of ages 45–68 years. Additionally, there was strong evidence of an effect of genetic liability on a lower hippocampal volume in the oldest age participants (aged 68–81 years), using genetic instruments both including and excluding the *APOE* locus. A study also using the UKB identified strong evidence of an effect of the AD PRS (p≤5×10^−8^) and hippocampal subfield volumes in older individuals (aged 63–80 years), which was driven by SNPs in the *APOE* locus.^51^ Contrary to previous PRS studies,^12 13^ we found weak evidence that genetic liability to AD was associated with a lower hippocampal volume in childhood. However, in comparison with the stringent threshold we used in our study (p<5×10^–8^), these studies used liberal p-value thresholds for SNP inclusion (p≤0.132 and p≤0.0001) (increasing risk of bias due to horizontal pleiotropy).^12 13^

The focus of previous PRS studies with brain MRI data on the hippocampus and the neocortex can be attributed to their well-recognised role in cognition and episodic memory.^53 54^ However, there are other structures that are relevant for cognition that are less well studied in relation to genetic liability to AD,^55^ such as the thalamus. The medial temporal lobe connects to thalamic nuclei and the retrosplenial cortex, constituting the hippocampal-diencephalic system, whose integrity is important for normal episodic memory.^56^ In our study, we found the earliest, most robust effects of genetic liability to AD in the thalamus as early as childhood (aged 8–14 years) and in the caudate and accumbens from 60 years of age. A study investigating how the *APOE* genotype changes whole-brain large-scale structural networks in subjects with mild cognitive impairment,^57^ found *APOE* ε4 carriers showed pronounced atrophy in specific regions such as the thalamus and the hippocampus, both of which had strong structural covariance association with the left caudate nucleus. Furthermore, a longitudinal brain imaging study examining the effects of the *APOE* ε4 genotype found evidence of differences between carriers/non-carriers in rates of amyloid-β plaque accumulation across the adult lifespan only in the caudate at age 56 years and the putamen at age 63 years.^58^ *APOE* ε4 carriers showed accelerated rates of amyloid-beta deposition in the entorhinal cortex at age 68 years. We observed that the oldest participants (aged 68–81 years) with higher genetic liability to AD showed, on average, lower entorhinal thickness.

Like other studies, we also found causal effects of genetic liability to AD on larger thickness in the lateral occipital, which is consistent with two previous studies in healthy individuals where *APOE* ε4 carriers have a thicker occipital cortex in comparison with normal controls.^59 60^ The thickening of certain brain regions has been speculated to reflect brain swelling in response to glial activation in preclinical AD stages.^61^

Genetic liability to AD is hypothesised to affect brain structures through influencing neurodevelopment, resulting in structural differences in the brain which may increase tolerance to pathology (ie, altering brain reserve and increasing the age of disease onset), or by changing the rates or mechanisms of neurodegeneration.^3^ We observe an age-dependent decrease in the volume of structures such as the thalamus, caudate and accumbens in UKB participants. However, a longitudinal analysis would be required to test the variable neurodegeneration hypothesis and such a conclusion cannot be extrapolated from findings in cross-sectional data (as in our analyses). Walhovd *et al* examined the association between AD PRS and hippocampal volume in 1181 cognitively healthy people with a wide age range (4–95 years).^4^ They identified an effect of a higher AD PRS on reduced hippocampal volumes in young adults, which was consistent across age groups, suggesting the AD PRS results in an earlier onset of brain ageing instead of accelerated ageing through variable neurodegeneration. The MR of brain morphology on AD in our study provides little support for the notion that brain structure alterations change the risk for AD, except for a larger hippocampal volume increasing the risk for AD, which is contrary to most existing research.^4 62 63^ This hippocampus finding from our MR study may be due to chance, or due to the small number of SNPs used (n=6). It is unlikely that these effects are a result of pathways independent of hippocampal volume (ie, horizontal pleiotropy), as the MR estimators which relax the assumptions about instrument validity are consistent with the IVW method, and there was little evidence of heterogeneity or pleiotropy in the causal effect estimates. Although we found little evidence of effects of brain morphology on AD risk, we observed that AD genetic liability influenced the volume of the thalamus from childhood to adulthood, which suggests that initial thalamic reserve could potentially play a role in AD risk. However, given that this structure is not typically implicated in the earliest AD-related brain atrophy, it is possible that this is a chance finding reflecting variability around the null. The caudal anterior cingulate was observed to be associated with genetic effects in childhood but not in adulthood. However, a recent recall-by-genotype study also reported an effect in this region in adults of the PROTECT cohort.^64^ In summary, the fewer effects observed in childhood and early adulthood compared with those later in the life course may be due to developmental noise, or a greater effect of genetic variation on more biological pathways in older individuals. It is also possible that genetic effects become more pronounced later in the life course due to the accumulation of gene-environment interactions and/or potential epigenetic mechanisms. Future studies should seek to replicate this in large independent samples with repeated measures when more data become available.

The MR method requires that genetic variants must fulfil three key assumptions to be considered valid instrumental variables: (1) that the genetic variants are strongly associated with the exposure (relevance assumption), (2) that there is no confounding of the genetic instrument – outcome association (eg, by population stratification, or dynastic effects; the independence assumption), and (3) that the genetic instruments affect the outcome only through the exposure (exclusion restriction assumption). Only the first assumption can be tested with the use of statistical parameters indicating instrument strength (variants associated with the trait at genome-wide significance and/or F-statistics in our analyses >10). The independence and exclusion restriction assumptions are not testable but are falsifiable with sensitivity analyses. We adjusted our GWAS for ancestry-informative principal components to control for population stratification. We were unable to account for dynastic effects in this study, but future within-family MR study designs should look to examine this. For the exclusion-restriction assumption, sensitivity analyses were performed to examine potential bias due to horizontal pleiotropy. That said, several brain measures had too few genetic proxies for pleiotropy sensitivity analyses to be performed and hence, these results should be considered with caution.

While previous studies have examined whether genetic liability to AD is associated with specific structural brain measures, our study is the first to examine these in such large samples, using an exploratory approach from childhood to old age. One of the main strengths of our study is that genetic variants are subject to little measurement error, contrary to observational neuroimaging phenotypes, and can serve as unconfounded indicators of particular traits values.^17^ Furthermore, using aggregate PRS precludes the examination of key potential sources of bias such as horizontal pleiotropy, which we have examined in detail here. We examined regions that have not been shown to be vulnerable to AD pathology, allowing us to discover novel regions affected by genetic liability to AD, such as the caudate. The large modern biobanks with neuroimaging and genetic data allowed us to recreate to the best of our ability a pseudo-longitudinal cohort. The precision of age-dependent dose effects suggest that our results are unlikely to be due to chance or other forms of bias. However, for studies such as ALSPAC, participants were selected for imaging for (1) a case-control study of psychotic experiences, (2) recall-by-genotype for schizophrenia, (3) testosterone study, making the ALSPAC sample unrepresentative of the general population. Another limitation is that different Freesurfer versions were used across cohorts. However, we allowed for this technical variation using random-effects meta-analyses. Although we applied multiple correction strategies controlling the false discovery rate, our findings were consistent across multiple cohorts. Finally, the participants in our analyses were of European ancestry and the findings may not be generalisable to other populations.

Our study shows that genetic liability to AD is associated with age-dependent changes in brain morphology in non-clinical populations, starting as early as 60 years of age, potentially highlighting the earliest phenotypic manifestations of the disease and the optimal timing for intervention with any potential neuroprotective therapy. Brain imaging to detect AD focuses on hippocampal, whole brain and parietal volume. The findings of our study highlight the importance of brain volume in other regions — notably the striatum — for AD. The analysis of these regions could be incorporated into early diagnosis imaging analysis algorithms for clinical use. The lack of evidence to support an effect of brain morphology on AD suggests that genetic liability to AD affects biological pathways leading to neurodegeneration rather than neurodevelopment. Future research should aim to use a longitudinal design and integrate their findings with biological and clinical data.

## Supplementary material

10.1136/jnnp-2023-332969 online supplemental table 1

10.1136/jnnp-2023-332969 online supplemental file 1

## Data Availability

Data may be obtained from a third party and are not publicly available.
